# Efficient Diode Performance with Improved Effective Carrier Lifetime and Absorption Using Bismuth Nanoparticles Passivated Silicon Nanowires

**DOI:** 10.3390/nano12213729

**Published:** 2022-10-24

**Authors:** Mariem Naffeti, Mohamed Ali Zaïbi, Alejandro Vidal García-Arias, Radhouane Chtourou, Pablo Aitor Postigo

**Affiliations:** 1 Laboratory of Nanomaterials and Systems for Renewable Energies (LaNSER), Research and Technology Center of Energy, Techno-Park Borj-Cedria, Tunis 2050, Tunisia; 2 Instituto de Micro y Nanotecnología, IMN-CNM, CSIC (CEI UAM+CSIC), Isaac Newton, 8, 28760 Madrid, Spain; 3 The Higher National Engineering School of Tunis, Tunis University, 5 Avenue Taha Hussein, Tunis 1008, Tunisia; 4 The Institute of Optics, University of Rochester, Rochester, NY 14627, USA

**Keywords:** Si nanowires, Bi nanoparticles, surface passivation, Schottky diode, minority carrier lifetime, absorption

## Abstract

In this paper, we report a novel design of bismuth nanoparticle-passivated silicon nanowire (Bi@SiNW) heterojunction composites for high diode performances and improved effective carrier lifetime and absorption properties. High-density vertically aligned SiNWs were fabricated using a simple and cost-effective silver-assisted chemical etching method. Bi nanoparticles (BiNPs) were then anchored in these nanowires by a straightforward thermal evaporation technique. The systematic study of the morphology, elemental composition, structure, and crystallinity provided evidence for the synergistic effect between SiNWs and BiNPs. Bi@SiNWs exhibited an eight-fold enhancement of the first-order Raman scattering compared to bare silicon. Current–voltage characteristics highlighted that bismuth treatment dramatically improved the rectifying behavior and diode parameters for Bi-passivated devices over Bi-free devices. Significantly, Bi wire-filling effectively increased the minority carrier lifetime and consequently reduced the surface recombination velocity, further indicating the benign role of Bi as a surface passivation coating. Furthermore, the near-perfect absorption property of up to 97% was achieved. The findings showed that a judicious amount of Bi coating is required. In this study the reasons behind the superior improvement in Bi@SiNW’s overall properties were elucidated thoroughly. Thus, Bi@SiNW heterojunction nanocomposites could be introduced as a promising and versatile candidate for nanoelectronics, photovoltaics and optoelectronics.

## 1. Introduction

Electronic and photovoltaic (PV) devices are in the spotlight worldwide both at the scientific and technological level where they are an integral part of our day-to-day life. Crystalline silicon (c-Si) is a cornerstone semiconductor material for the electronic and PV industries. This prominent position hails from a unique set of beneficial features: non-toxic and environmentally friendly; high purity and stability; high carrier mobility; effective conductivity engineering; superior thermal and mechanical properties; well-established fabrication approaches and, crucially, naturally abundance as the second most abundant element on earth [1,2,3].

Despite the positive attributes, the optical properties of c-Si are relatively poor, due to its indirect bandgap, which hinders the efficient emission and absorption of light. Besides, the high-cost of Si wafers restricts its cost-competitiveness, engendering a need for an alternative type of thinner Si wafers with reduced costs and performance improvements. Because of this, novel Si nanostructures have been fabricated and demonstrated over the years, including silicon nanotubes [4], silicon nanowires [1,2,3,5], silicon nanocones [6,7], silicon nanorods [8], silicon nanoholes [9,10], silicon nanocrystals [5,11] and silicon nanopillars [12,13].

Of particular interest are the silicon nanowires (SiNWs), which provide unique advantages in terms of strong light absorption and efficient charge separation and collection owing to their large surface area, thus facilitating its implementation in various optical energy harvesting devices in optoelectronic, photonic and photovoltaic applications [1,2,3,5]. A variety of physical and chemical methods are currently available to synthesize SiNWs [1,2,3,14]. Among others, metal-assisted chemical etching (MACE) allows several advantages as it is inexpensive, simple, controllable, and can handle large-quantitative production.

Although steady progress on SiNW-based devices has been made in recent years, an ongoing challenge persists towards realizing highly efficient devices owing to the large amounts of impurities, dangling bonds, flaws generated during the fabrication process, spontaneous oxidation in ambient atmospheres as well as recombination activities. These adversely alters device performance and reliability and hinders their practical usage in commercial products. Because of this, surface passivation is necessary to remedy such issues and enhance device performance. A significant level of surface passivation often fulfills three major tasks: it satisfies undesirable dangling bonds at the surface of the underlying Si nanostructure, thus minimizing the number and/or the recombination of interface trap states; it provides a fixed electric field that repels carriers, curbing its recombination; and it reduces the optical loss or enhances light absorption.

Indeed, many SiNW passivation attempts have been reported in the literature, and various materials have been used. These materials include semiconductor nanoparticles, polymers, oxides, metal nanoparticles, etc. [15,16,17,18,19,20]. The developed material@SiNW compounds exhibit remarkably enhanced device performances compared to pure SiNWs. For instance, an enhancement in current (I) by over three orders of magnitude was recently reported for ZnO@SiNW heterojunctions [15]. Chiou et al. demonstrated an enhanced rectification behavior for TiO_2_@SiNW heterojunctions [16]. Amri et al. reported a significant decrease and increase in the series resistance and rectification ratio, respectively, for Li@SiNW heterojunctions [17]. The study of Zaïbi and her co-workers revealed a substantial enhancement of the electro-performance of functionalized SiNWs by a conductive polymer coated with gold nanoparticles [18]. Other studies have shown an improvement in the ideality factor from 8.85 (unmodified SiNWs) to 7.78 for Ag@SiNW heterojunctions [19]. An improved power conversion efficiency of 9.36% has been achieved recently after inserting an Al_2_O_3_ passivation layer on to SiNWs [20].

Far afield of these relatively expensive materials and to our best knowledge, bismuth (Bi) (which has been recognized as ‘the wonder metal’) is a favorable and advantageous option that has not yet been explored for the passivation of SiNWs to enhance the electronic and photovoltaic performance. This post-transition metal is very promising for several essential applications, including biomedicine, telecommunication, light-emitting diodes, electronics and solar cells since, apart from being abundant, inexpensive, and environmentally friendly, it is a good absorber and possesses plasmonic properties [21,22,23,24].

In the present work, we find a way to prepare Bi-modified Si nanowire composites (Bi@SiNWs). We assessed the passivation capabilities of Bi coating on to SiNWs for reliable and performant heterojunction diodes with improved carrier lifetime and optical properties. The obtained products were characterized using SEM, EDX, DRX, XPS, and Raman analysis. The electrical properties and parameters of Ag/SiNWs-Si/Al and Ag/Bi@SiNWs-Si/Al heterojunction diodes were carefully determined and studied. The minority carrier recombination lifetime and absorption measurements were investigated. Overall improvement of Bi-passivated devices performance was explored, highlighting its potential use in electronic, optoelectronic and photovoltaic applications.

## 2. Materials and Methods

SiNWs were synthesized from p-type silicon substrate (orientation: (100); resistivity: 1–20 Ωcm; and thickness: 500 µm) via silver-assisted chemical etching (Ag-ACE). The substrates were first ultrasonically cleaned in acetone, ethanol, isopropanol and de-ionized water (DI) for 15 min each and then immersed in diluted HF for 3 min. The cleaned substrates were dipped into a solution containing 4.8 M HF and 0.035 M AgNO_3_ for electroless deposition of silver nanoparticles (AgNPs) for 1 min. the Ag-loaded SiNWs were then immersed into the etching solution of 4.8 M HF and 0.5 M H_2_O_2_ at room temperature for 20 min. The resulting surfaces were soaked in HNO_3_ solution for 15 min to remove the residual AgNPs. The formed black SiNWs were ultimately washed with DI and dried in nitrogen.

Subsequentially, Bi@SiNW composites were prepared by decoration of the as-prepared SiNWs with BiNPs through thermal evaporation of high-purity metal Bi powder. The evaporation started at a base pressure of 10^−5^ torr with a constant rate of 0.5 A°s^−1^. A thickness series was deposited on to SiNW surfaces (T_1_ = 5 nm, T_2_ = 20 nm and T_3_ = 35 nm) which were controlled through quartz crystal microbalance. The final products were annealed at 100 °C for 15 min under a nitrogen atmosphere and marked as “Bi-1@SiNWs”, “Bi-2@SiNWs” and “Bi-3@SiNWs” (corresponding to T_1_, T_2_ and T_3_, repectively). Figure S1 shows a scheme of the fabrication process.

The samples were characterized by scanning electron microscopy (SEM, FEI Varios 460, FEI Europe B.V., Eindhoven, The Netherlands), energy dispersive X-ray spectra (EDX) attached to the SEM and X-ray diffraction (XRD, Bruker D8 advance, Cu Kα radiation, λ = 1.54 Å). X-ray photoelectron spectroscopy (XPS) was performed using a SPECS GmbH system (SPECS Surface Nano Analysis GmbH, Berlin, Germany). Raman spectra were obtained by using a micro-Raman spectrometer (Jobin–Yvon LabRAM HR 800 UV, Horiba, Edison, NJ, USA) with a 633 nm He–Ne laser as the excitation light and fixed at a power of ~0.03 mW. The electrical measurements were conducted via a Keithley 2400 source meter (Tektronix, Inc., Beaverton, OR, USA) in the dark and at room temperature. A Semilab WT-2000 PVN was employed for minority carrier lifetime mapping via the microwave photoconductivity decay (µPCD) technique (λ = 905 nm, F = 10 GHz). The absorption spectra of the prepared samples were assessed by measuring the spectral reflectance and transmittance via a Perkin Elmer Lambda 950 spectrophotometer (Perkin Elmer, Llantrisant, UK).

## 3. Results and Discussion

**Figure 1:**
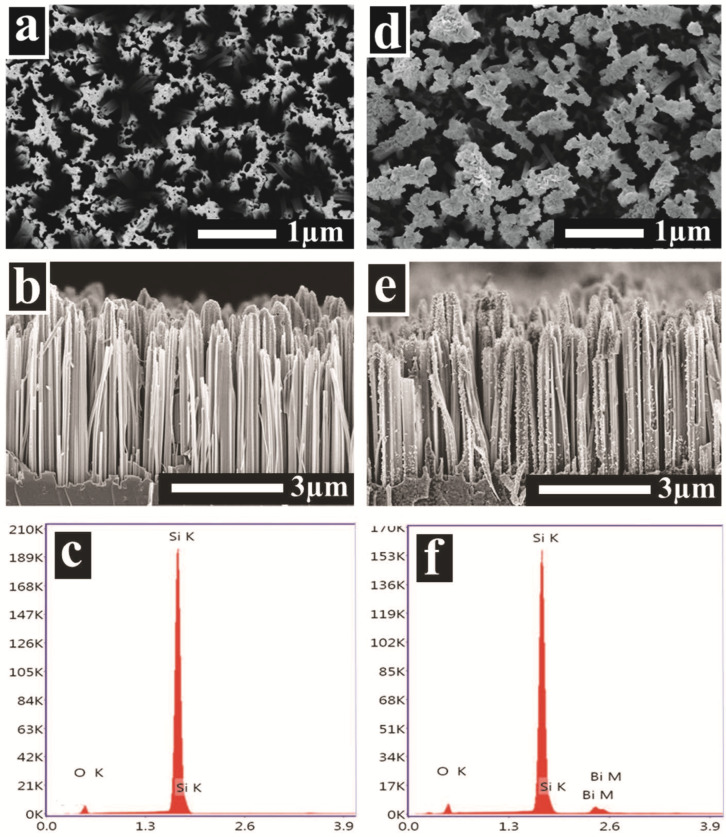
Top-view SEM images of SiNWs: (**a**) before and (**d**) after BiNPs decoration (Bi-3@SiNW sample). (**b**,**e**) corresponding cross-sectional images and (**c**,**f**) corresponding EDX spectra.

Surface morphology of SiNWs and Bi@SiNWs was studied by SEM. Forest-like SiNW arrays were observed from the top-view image (Figure 1a) where the NWs aggregated together to form bundles owing to van der Waal attraction [3]. The SiNWs with a length and diameter of about 5 µm and 100~200 nm, respectively, were observed to be vertically aligned, as displayed in Figure 1b. EDX spectra shown in Figure 1c revealed that the SiNWs consist of silicon and oxygen. After Bi incorporation, BiNPs were discernible along the whole of the SiNWs from the top (Figure 1d) to the sidewall and bottom (Figure 1e). The corresponding EDX spectra of Figure 1f corroborates the presence of Bi.

Figure S2 in Supplementary Materials further shows the BiNPs distribution and their elemental concentration.

**Figure 2:**
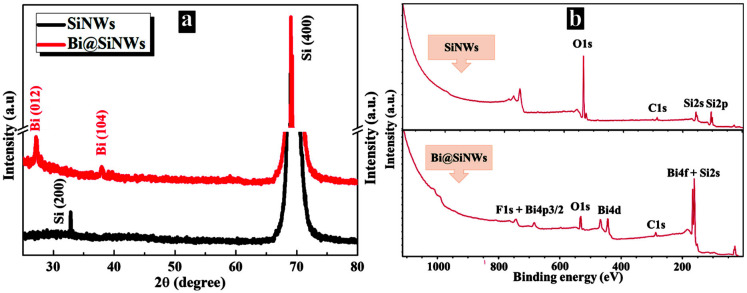
XRD patterns (**a**) and XPS survey spectra (**b**) of SiNWs and Bi@SiNWs.

The crystal structure of the prepared samples was investigated through XRD measurements. As evidenced from Figure 2a, both the pure SiNWs and Bi-modified SiNWs demonstrate well-formed crystalline silicon structures with a characteristic peak at 69°, assigned to the (400) crystal plane of Si. The extra peak appearing at 33° in the SiNWs XRD pattern belongs to the Si (200) plane. On the other hand, two additional peaks located at 27° and 38° appeared in the XRD pattern of the Bi@SiNW composite. These peaks are related to Bi (012) and Bi (104), respectively [25,26].

To further probe into the interaction between BiNPs and SiNWs in the as-synthesized composite, XPS measurements were carried out. As shown in Figure 2b, the XPS Si2s, Si2p, O1s, and C1s peaks agree with the chemical composition of the wired silicon. On the other hand, one can see the appearance of new peaks in the Bi@SiNW survey, which corresponds to 5p, 4f, 4d, and 4p3/2 of Bi signals [25]. The deconvolution of the Si2s and Bi4f peaks are shown in Figure S3. All the above results are indicative of the successful synthesis of a Bi@SiNW nanocomposite.

**Figure 3:**
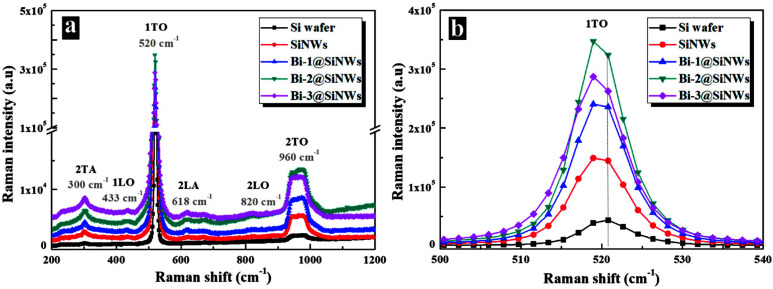
(**a**) Raman spectra of bulk Si, SiNW and Bi-modified SiNW samples. The curves are slightly shifted vertically to enable comparison. (**b**) Comparison of the first-order Raman spectra for different samples.

Another important aspect of our investigation was the study of enhanced Raman scattering from Bi nanoparticle-coated SiNW samples. For comparison, the blank Si substrate and pure SiNWs were also studied, and the results are shown in Figure 3.

The Raman spectra from the c-Si display a symmetric Lorentzian Raman peak located at 520.63 cm^−1^, which implies a first-order transverse optical mode (1TO) [27]. Enhanced Raman intensity of SiNWs was recorded compared to bare Si, indicating a good crystallinity entirely consistent with the XRD results [28]. This behavior could be related to an increase in light and matter interaction and hence a higher Raman scattering intensity [28,29]. This is compatible with the optical absorption results, as discussed later. The scattering intensity enhancement has been previously observed in silicon nanostructures [28,30,31]. According to the literature, the 1TO Raman peak of SiNWs is slightly downshifted (red-shift) and broader due to the quantum confinement effect on phonons [28,32].

Encouragingly, BiNP deposition has been shown to cause an increase in the main peak intensity by a factor of 8. To explain this amplification, we referred to the work of Rani et al. in which they discuss the role played by silver nanoparticles in the enhancement of the first-order Raman mode of SiNWs [32]. It was observed that the signal enhancement was mainly attributed to the plasmonic effect, which is generated locally between metal nanostructures. Consequently, surface-enhanced Raman spectrum can be observed here. Furthermore, several multi-phonon peaks and bands, ranging from 300 to 960 cm^−1^, are present in all Raman spectra, corresponding to the 2TA, 1LO, 2LA, 2LO and 2TO modes [27,29,33]. It is noteworthy that all the spectra are similar, with an increase in peak and band intensities after SiNW formation and Bi loading.

**Figure 4:**
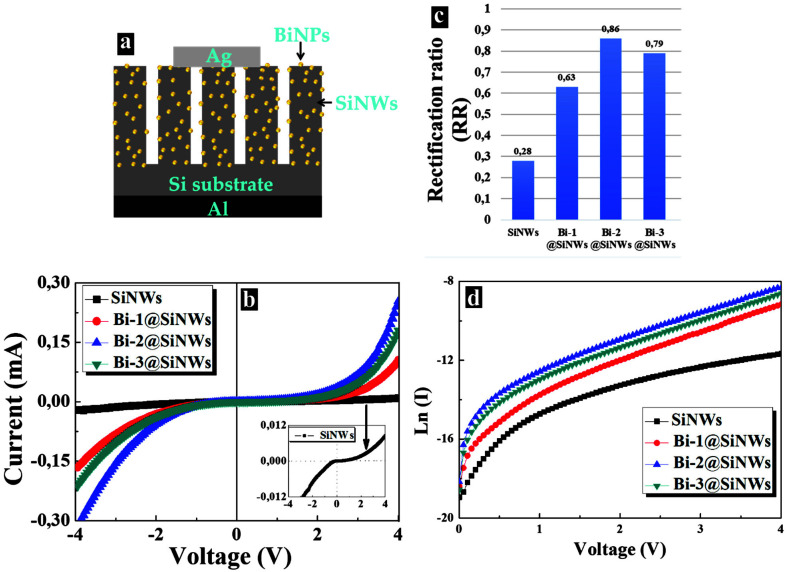
(**a**) Schematic illustration of the Ag/Bi@SiNWs-Si/Al device. (**b**) I–V characteristics of the prepared device at different Bi thicknesses compared to a Bi-free device. The inset displays the enlarged I–V curves of the Bi-free device. (**c**) Comparison of the devices rectification ratio (RR). (**d**) Ln (I) vs. V curves of the prepared devices.

The electrical properties were studied by current–voltage (I–V) characteristics in order to examine the effect of SiNWs decorated with Bi of different thicknesses (T_1_, T_2_, and T_3_) on the electrical response. The metal contacts on the front and the rear were formed using silver and aluminum, respectively. Figure 4a displays a schematic illustration of the fabricated Ag/Bi@SiNWs-Si/Al heterostructure and the resulting I–V measurements at room temperature in darkness are depicted in Figure 4b.

It is evident from these curves that Bi incorporation significantly improves the I–V response. Precisely, all the devices with NWs treated with Bi indicated a superior diode rectification behavior, whereas the untreated SiNW devices indicated poor junction characteristics where only several µA of current could be detected, as shown in the inset of Figure 4b.

From here, the rectification ratio (RR) was found to increase after Bi treatment (Figure 4c). Accurately, the RR is defined as the ratio magnitude of the current for positive (forward) and negative (reverse) voltages at ±4 V; (RR(V) = $\frac{I\left(V\right)}{I\left(-V\right)}$).

The nonlinear I–V responses and the obtained rectifying event for both Bi-free and Bi-modified devices, regardless of the extent, indicated the formation of Schottky barriers at the Ag/SiNWs-Si and Ag/Bi@SiNWs-Si interfaces, respectively, since the Si/Al interface has been proven to be ohmic [3,34]. The rectification in the unmodified device is due to the quantum confinement coming from the silicon nanocrystals in the SiNWs, which increases the band-gap on the SiNW side and thus creates a potential barrier resulting in the observed rectifying property [3,35].

On the other hand, for Bi modified devices, the introduction of Bi catalyst atoms caused an effective n-type doping in SiNWs to Si [36,37,38]. This established a depletion layer and subsequently caused the observed rectifying nature. Moreover, the SiNWs treatment with Bi offered superior surface passivation, leading to an overall characteristic performance enhancement. Accurately, Bi treatment led to a reduction in the defect traps density and quenched a large part of the Si dangling bonds produced during the etching process of SiNW formation. Thus, a reduction in the recombination activities occurred. Furthermore, the embedding of Bi plasmonic nanoparticles within the structure of the SiNWs might increase the mobility of charge carriers. Consequently, we can assume that the different effective Schottky barriers mainly refer to the differences in the I–V behaviors

In order to further evaluate the improvement of Schottky junction quality, we determined the devices electrical parameters using the conventional thermionic emission model (TE) as expressed:

(1) $$I=I_{s}\left(\exp\left(\frac{q\left(v-{\mathrm{IR}}_{s}\right)}{\eta \mathrm{kT}}\right)-1\right)$$

where q is the electronic charge, η is the ideality factor, R_s_ is the series resistance, k is the Boltzmann constant, T is the absolute temperature and I_s_ is the reverse saturation current given by:

(2) $$I_{s}={\mathrm{aA}}^{*}T^{2}\exp\left(\frac{-{q\varphi }_{b}}{\mathrm{kT}}\right)$$

where a is the diode area, A* is Richardson constant (≈32 A cm^−2^ K^−1^ for p-type Si) and $\varphi_{b}$. is the barrier height. As eV >> ηKT at room temperature, Equation (1) can be rewritten as:

(3) $$I=I_{s}\exp\left(\frac{q\;V}{\eta \mathrm{kT}}\right)\;$$

The ideality factor η was estimated from the slope of the linear region of the plot of ln(I) vs. V (Figure 4d) based on Equation (3). The saturation current was derived by extrapolating ln(I) vs. V plot to V = 0, while $\varphi_{b}$ was calculated through Equation (2).

To have more accuracy in the ideality factor values of the non-linear part of ln(I) vs. V and for the determination of the series resistance (R_s_), the Cheung’s functions were utilized [19,34]. Thus:

(4) $$\frac{\mathrm{dV}}{d\left(\mathrm{LnI}\right)}=R_{s}I+\frac{\eta \mathrm{kT}}{q}$$

**Figure 5:**
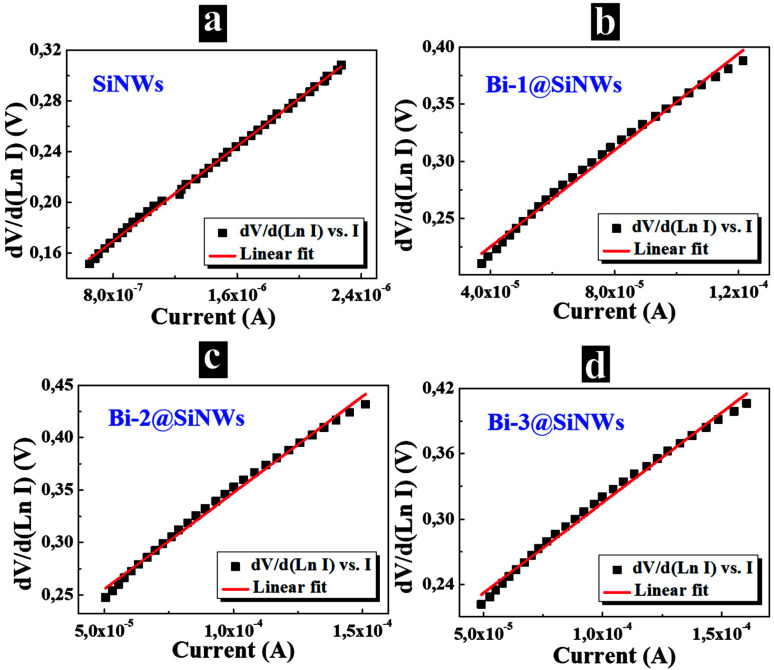
dV/d(LnI) vs. I plots of the prepared devices. (**a**) SiNWs-based device. (**b**) Bi-1@SiNWs-based device. (**c**) Bi-2@SiNWs-based device. (**d**) Bi-3@SiNWs-based device.

Figure 5a–d show the experimental $\frac{\mathrm{dV}}{d\left(\mathrm{LnI}\right)}$ vs. I plots, the R_s_ and η were determined as the slope and *y*-axis intercept, respectively.

The deduced values of I_s_, η, $\varphi_{b}$ and R_s_ are summarized in Table 1. For the Bi-free device, η and $R_{s}$ values were found to be relatively high owing to a large amount of surface defects and dangling bonds, indicating quite poor junction quality. After Bi decoration, one can note a notable improvement in the device’s performance and a dependence on Bi thickness. Indeed, η and R_s_ exhibited decreased values’ due to (i) a superior surface and interfacial passivation effect through reducing the abundant dangling bond and surface defects and (ii) the reduction of the surface recombination activities. The latter will be further discussed in the minority carrier lifetime part. When exceeding T_2_ moderated thickness, an aggregation of excessive Bi atoms led to the formation of a resistive region which explains the slight deterioration of the device’s characteristics.

It is worth noting that these η values are lower compared to those reported in other modified SiNW devices [19,39,40,41,42] (Table S1). A similar trend was noticed for I_s_ and $\varphi_{b}$. The ideality factor values estimated from both Cheung’s functions and I–V method matched considerably. These above-mentioned results prove a successful implementation of an efficient device based on SiNWs decorated with a moderate thickness of Bi. They, therefore, could be considered as a promising candidate in electronic devices and PV.

To better scrutinize the passivation of SiNWs with the BiNPs, we measured the minority carrier lifetime (τ). This is an ideal parameter for inline material quality and process control characterization. Accurately, high τ indicates material of good quality, while low τ denotes problems, namely, the presence of impurities, dislocations, and defects in the semiconductor material. Thus, τ is of essential importance for the performance of various semiconductor devices.

**Figure 6:**
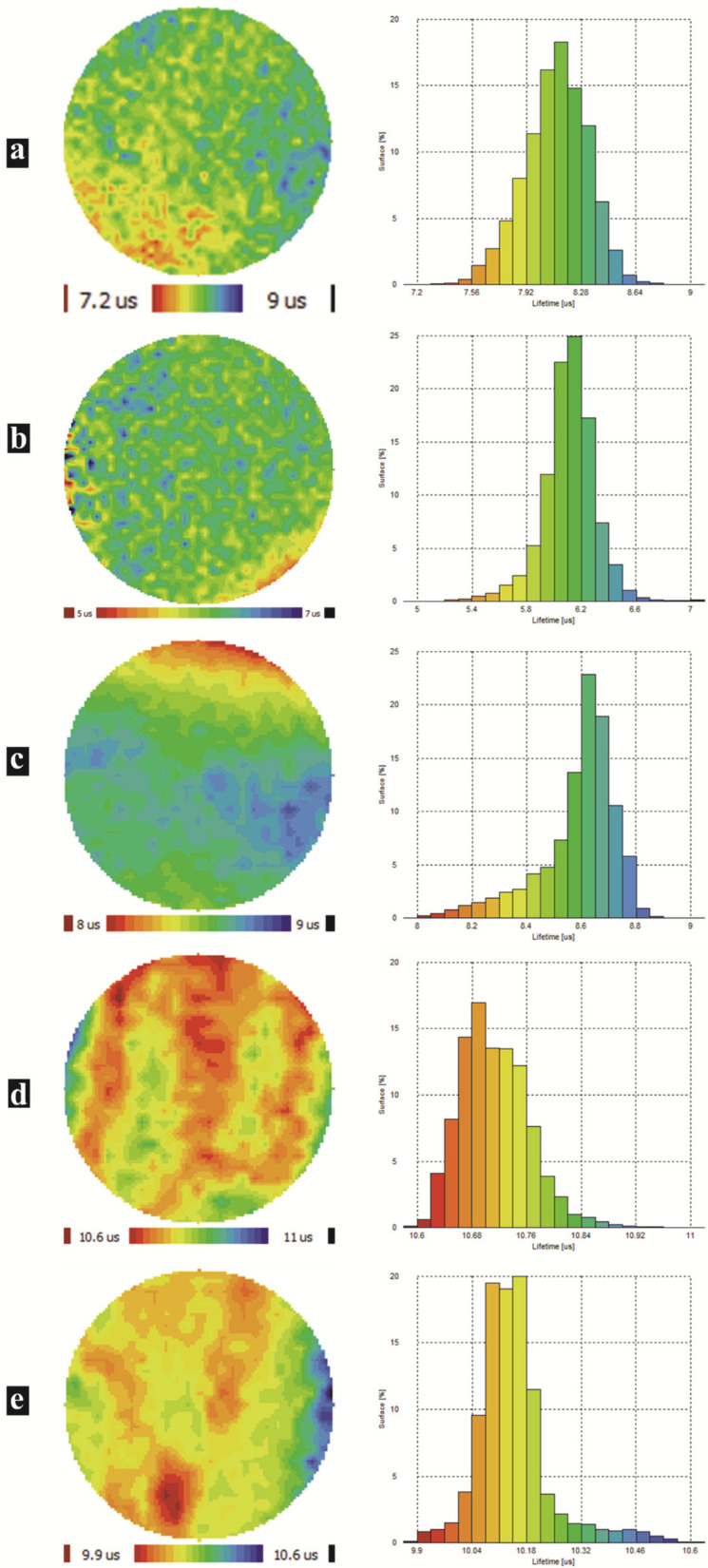
Effective lifetime maps and corresponding histograms of (**a**) bare silicon, (**b**) pure SiNWs, (**c**) Bi-1@SiNWs, (**d**) Bi-2@SiNWs and (**e**) Bi-3@SiNWs.

Figure 6 displays the lifetime maps and the corresponding histograms. The sample series included a bare silicon substrate as a reference, pure SiNWs, and Bi-coated SiNWs with different Bi thicknesses.

The experimentally measured value of τ refers to the devices effective lifetime (τ_eff_) and is defined as the average time it delays an excited minority carrier (electrons in p-type Si) to recombine with a majority carrier (holes in p-type Si). The τ_eff_ average values were extracted from the lifetime mapping. One can note that we took into account the middle of the samples, where there was the most homogeneity and uniformity, and excluded the borders, where there were the cleavage marks. The efficiency of the surface passivation was quantified via the effective surface recombination velocity (S_eff_) determination from the following expression [17,40]:

(5) $$\frac{1}{\tau_{\mathrm{eff}}}=\frac{1}{\tau_{b}}+\frac{2s_{\mathrm{eff}}}{w}$$

where τ_b_ is the bulk recombination lifetime, and w is the wafer thickness. One can assume a high value of the bulk lifetime and then most of the recombination activities occur on the wafer surfaces. Hence, the upper limit of S_eff_ was derived from Equation (5) [17]:

(6) $$\mathrm{Seff}\leq \frac{w}{2\tau_{\mathrm{eff}}}\;$$

Table 2 summarizes the τ_eff_ and S_eff_ values. The original polished Si wafer exhibited a lifetime and surface recombination velocity of about 8.1 µs and 3086.4 cm.s^−1^, respectively.

After the formation of large-scale vertical SiNWs, a deterioration of both τ_eff_ and S_eff_ occurred. This behavior denotes a relatively low SiNW surface quality, due to the generation of dangling bonds and surface defects during the Ag–ACE process. The latter increases deep-level traps, leading to severe surface recombination. Therein lies the importance of surface passivation as a powerful tool that ensures the reduction of such recombination. Effectively, once the Bi treatment was established, we observed a net enhancement in the τ_eff_ and consequently in the S_eff_. The optimal passivation was obtained from the Bi-2@SiNW nanocomposite with τ_eff_ as high as 10.7 µs and S_eff_ as low 2336.4 cm·s^−1^.

These improvements originated from the saturation of dangling bonds and the minimization of defect traps through Bi anchoring. Thereby, a decrease in the recombination activities occurred, causing an enhancement in the surface quality of the SiNW arrays. However, one can see a slight dwindling of the lifetime for Bi-3@SiNWs, but it remains more intense than that of the untreated Si wafer. This relatively low passivation quality mainly owes to the generation of lifetime killer centers by forming large Bi nanoclusters that eventually aggregate together to create a thick Bi layer. This supports the electrical findings on the one hand and, on the other hand, reinforces that the passivation quality is highly susceptible to the amount of bismuth, which entails judicious optimization. In brief, our results strongly show how Bi treatment can assist in the development of improved SiNW surface quality. This brings to light the effectiveness of bismuth as a passivation material and opens up the possibility of using the Bi@SiNW nanocomposite for photovoltaic and optoelectronic applications.

**Figure 7:**
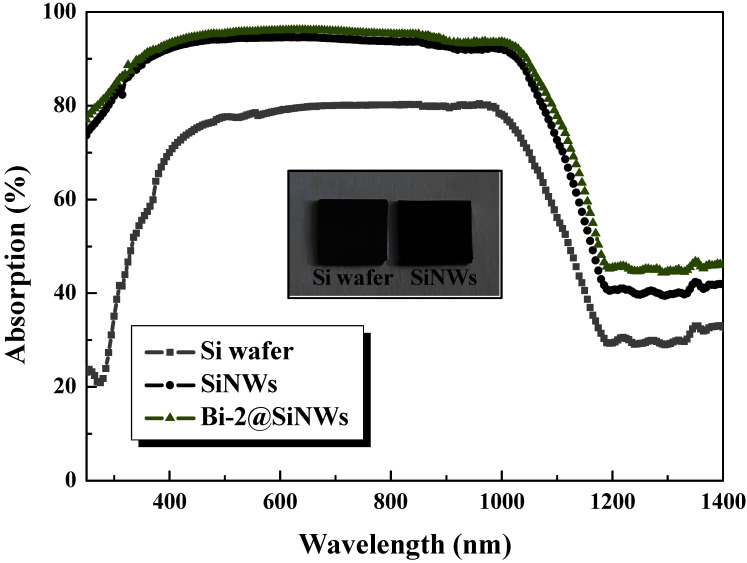
Absorption spectra of silicon wafers, sole SiNW and Bi-2@SiNW compounds.

It is interesting to assess further the integration effectiveness of Bi@SiNW nanocomposites in photovoltaics and other related electronic devices. To this end, measurements of one of the main optical parameters, i.e., the absorption (A), were performed. The spectral absorption of the prepared samples was evaluated by measuring the corresponding spectral reflection (R) and transmission (T) according to R(%) + T(%) + A(%) = 100%. The measurements were carried out over the wavelength range of 250–1400 nm, corresponding to the high spectral irradiance of sunlight, which is vital for silicon-based solar cells. The absorption spectra of the samples are shown in Figure 7.

The commercial bare silicon substrate exhibited low absorption, ranging from 21% to 79% in the UV–visible range. These relatively demoted values are due to the smooth silicon surface, free of incident light-trapping structures. After realizing a large-scale of 5 μm vertical SiNWs, the amount of absorbed light was significantly increased to above 93% in the entire silicon absorbing region (Si opaque region). This is ascribed to the ideal tapered architecture of silicon wires and to the gradient of the refraction index between air and the SiNW [43]. Thus, a strong light field confinement effect occurred, supported by the black and dull surface appearance of the SiNWs against the gray and reflective Si wafers, as displayed in the inset of Figure 7.

Higher net internal absorption over 97% was obtained via Bi@SiNW nanocomposites. This spotlights the effectiveness of bismuth as an anti-reflective coating and in preserving the optical properties of SiNWs. The reasons behind the increase in absorption are the enhancement and lengthening of the optical path, in addition to the effect of localized surface plasmonic resonance of the semimetallic Bi. These combined effects thus increase the capture ratio of photons. One can be observed that the sub-bandgap absorption, i.e., in longer wavelengths where Si is transparent, decreased due to light scattering [44]. In short, this high-absorption capability of both SiNWs and Bi@SiNWs will substantially strengthen the light harvesting in solar cells and other related optoelectronic devices.

## 4. Conclusions

In summary, we unveiled a simple and effective method to synthesize bismuth-passivated silicon nanowire heterojunction composites, offering high diode performances, and an improved effective carrier lifetime and absorption properties. The SiNWs were obtained through the Ag-ACE process, while thermal evaporation was used to deposit BiNPs on to the wires. The vertically aligned NWs acted as good anchors for the attachment of BiNPs. The resultant nanostructures were characterized from the point of view of morphology, elemental composition, structure and crystallinity properties, providing evidence for a strong interaction between SiNWs and BiNPs. Enhanced first-order Raman scattering by a factor of 8 was obtained compared to a planar Si sample. The electrical properties of Bi-free and Bi-passivated SiNW heterojunctions were studied through I–V measurements. The diode parameters were determined from conventional thermionic emission and Cheung’s model. When compared to Ag/SiNWs-Si/Al Schottky barrier diode, Ag/Bi@SiNWs-Si/Al showed superior diode rectification behavior and a substantial enhancement in the overall device’s performance, such us remarkably reduced η and Rs. Furthermore, Bi@SiNW heterojunctions exhibited an increase in the minority carrier lifetime and, subsequently, a decrease in the surface recombination velocity. Robust light-absorption of up to 97% was also achieved. The outcomes validate the potent role of Bi as a surface passivation coating; however, only at optimized amounts. Various factors are behind the superior improvement of Bi@SiNW’s overall properties and have been elucidated thoroughly. Our Bi@SiNW heterojunction nanocomposites are multifunctional and show promise for application in electronic, photovoltaic, and optoelectronic fields.

## Figures and Tables

**Table 1:** Electrical parameters determined by conventional TE and Cheung’s model.

	Ln (I) vs. V (TE Model)		Cheung’s Functions		
	I_s_ (µA)	η	φ_b_ (eV)	R_S_ (kΩ)	η
SiNWs	0.21	5.91	0.806	93.65	3.65
Bi-1@SiNWs	0.85	3.06	0.770	14.06	2.85
Bi-2@SiNWs	1.94	1.88	0.748	8.25	1.96
Bi-3@SiNWs	1.33	2.58	0.758	10.19	2.29

**Table 2:** Effect of Bi-passivation on τ_eff_ and S_eff_ of SiNWs.

Samples	Effective Minority Carrier Lifetime τ_eff_ (µs)	Effective Surface Recombination Velocity S_eff_ (cm·s^−1^)
Si	8.1	3086.4
SiNWs	6	4166.6
Bi-1@SiNWs	8.6	2906.9
Bi-2@SiNWs	10.7	2336.4
Bi-3@SiNWs	10.1	2475.2

## Data Availability

Not applicable. Funding M.N. and P.A.P. acknowledge the funding from CSIC under i-COOP+ program.

## Supplementary Materials

The following supporting information can be downloaded at: https://www.mdpi.com/article/10.3390/nano12213729/s1, Figure S1: Schematic illustration for the fabrication of BiNPs@SiNWs nanocomposite, Figure S2: (a,b) TEM images of Bi-rich SiNWs sample and (c) corresponding EDX table of the elemental concentration, Figure S3: Deconvolution of Bi4f and Si2s XPS peaks; Table S1: Comparison of Bi@SiNWs diode performance with other modified-SiNWs devices of various works.
