# Supplementary material for: Efficient Diode Performance with Improved Effective Carrier Lifetime and Absorption Using Bismuth Nanoparticles Passivated Silicon Nanowires
Source: Nanomaterials (Basel). 2022 Oct 24;12(21):3729. doi: 10.3390/nano12213729 (PMC9656161; doi:10.3390/nano12213729)
Supplement: Supplementary file 1 [file nanomaterials-12-03729-s001.zip › nanomaterials-1964956-supplementary/nanomaterials-1964956-supplementary.pdf]

Supplementary Materials

Article

Efficient Diode Performance with Improved Effective Carrier Lifetime and Absorption using Bismuth Nanoparticles Passivated Silicon Nanowires

Mariem Naffeti [1,2,3,] *, Mohamed Ali Zaïbi [1,3], Alejandro Vidal García-Arias [2], Radhouane Chtourou [1] and Pablo Aitor Postigo [2,4]

1 Laboratory of Nanomaterials and Systems for Renewable Energies (LaNSER), Research and Technology Center of Energy, Techno-Park Borj-Cedria, Bp 95, 2050 Hammam-Lif, Tunis, Tunisia
2 Instituto de Micro y Nanotecnología, IMN-CNM, CSIC (CEI UAM+CSIC) Isaac Newton, 8, E-28760 Tres Cantos, Madrid, Spain
3 Tunis University— The Higher National Engineering School of Tunis, 5 Av Taha Hussein 1008 Tunis, Tunisia
4 The Institute of Optics, University of Rochester, Rochester, New York 14627, USA

* Correspondence: naffeti.mariam@gmail.com

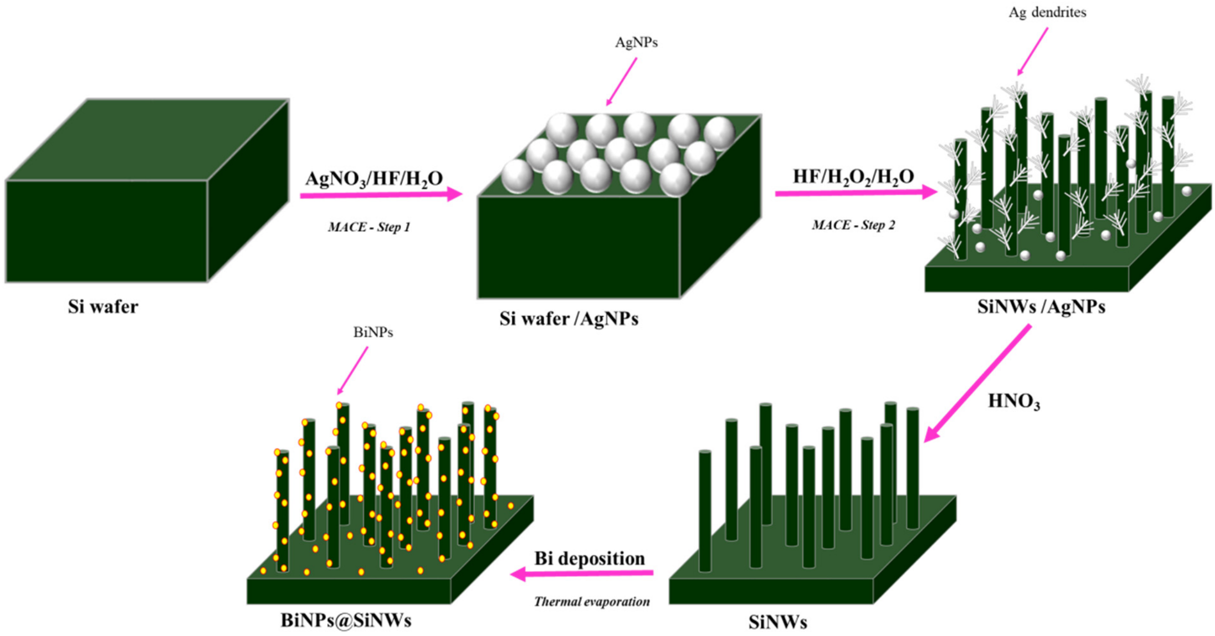

Figure S1. Schematic illustration for the fabrication of BiNPs@SiNWs nanocomposite

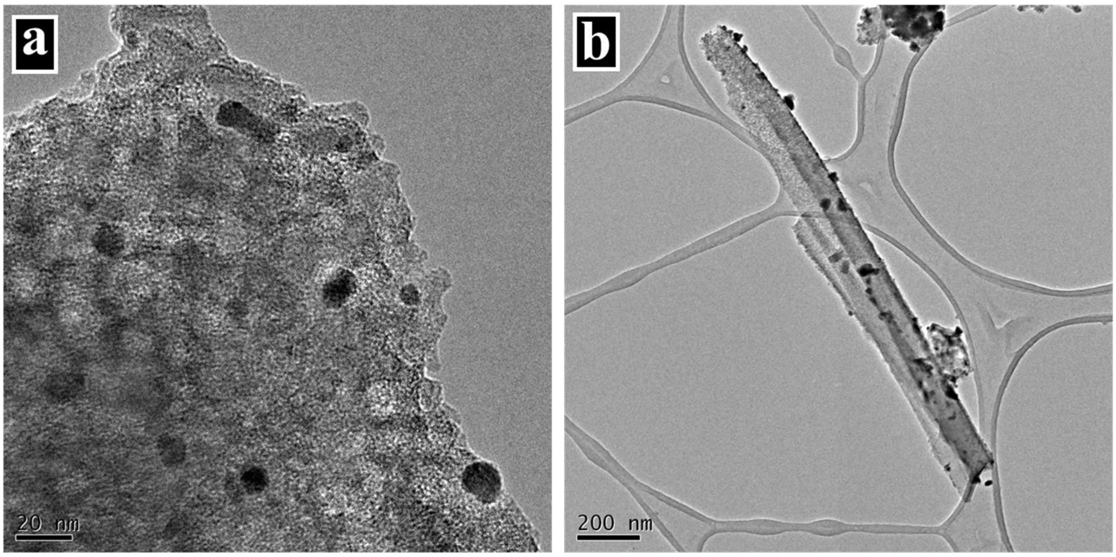

c

Element	Weight %	Atomic %	Net Int.	Error %	Kratio	Z	R	A	F
C K	9.04	18.33	40.76	12.08	0.0085	1.1327	0.9261	0.0833	1.0000
O K	9.44	14.38	229.55	9.70	0.0203	1.0868	0.9491	0.1979	1.0000
SiK	76.93	66.75	14355.20	1.75	0.7123	0.9923	1.0002	0.9314	1.0019
BiM	4.59	0.54	186.63	5.78	0.0272	0.6423	1.4068	0.8660	1.0679

Figure S2. (a,b) TEM images of Bi-rich SiNWs sample and **(c)** corresponding EDX table of the elemental concentration

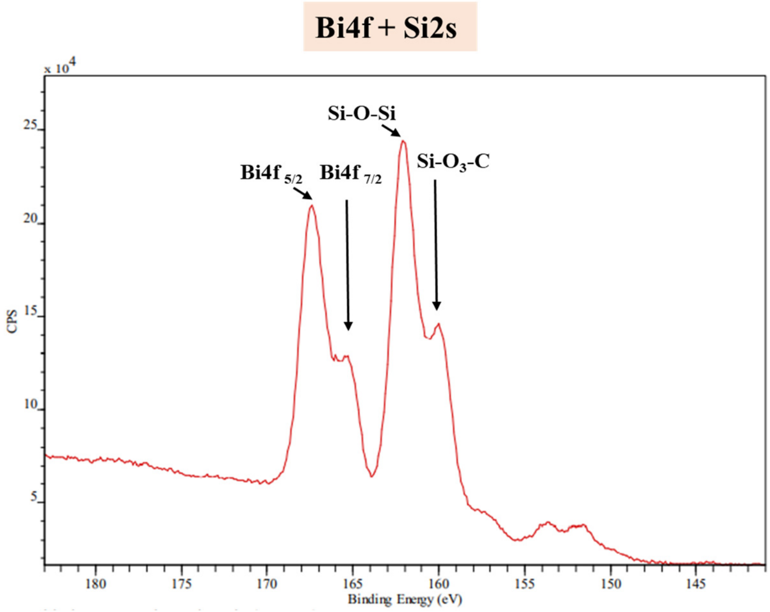

Figure S3. Deconvolution of Bi4f and Si2s XPS peaks

Table S1. Comparison of Bi@SiNWs diode performance with other modified-SiNWs devices of various works

Devices	SiNWs formation technique	Material deposition technique	η determination technique	η	references
**Pt@SiNWs**	MACE	Electroless deposition	conventional TE	9.61	[19]
**Ag@SiNWs**	MACE	Electroless deposition	conventional TE	7.78	[19]
**Graphene@SiNWs**	MACE	PECVD	conventional TE	3.92 ~ 5.52	[39]
**Ag@SiNWs/polymer**	MACE	Electroless deposition	conventional TE	2.16 ~ 2.41	[40]
**Pt@SiNWs**	MACE	Placing Pt Tip (monocontact)	conventional TE	3.7	[41]
**Polymer@SiNWs**	MACE	Spin coating	conventional TE	7.8	[42]
**Bi@SiNWs**	MACE	Thermal evaporation	conventional TE → Cheung's functions →	1.88 1.96	This work
